# Retraction: Specific PKC βII inhibitor: one stone two birds in the treatment of diabetic foot ulcers

**DOI:** 10.1042/BSR20171459_RET

**Published:** 2026-08-25

**Authors:** Sushant Kumar Das, Yi Feng Yuan, Mao Quan Li

**Affiliations:** 1 Department of Interventional and Vascular Surgery, Shanghai Tenth People's Hospital, Tongji University, 301 Yanchang Road, Shanghai 200072, China; 2 Department of Interventional Radiology, Affiliated Hospital of North Sichuan Medical College, 63 Wenhua Road, Nanchong, Sichuan 637000, China

**Keywords:** angiogenesis, diabetic foot, extracellular traps, NETosis, neutrophil, Protein Kinase C

This article is being retracted from *Bioscience Reports* at the request of the Editor-in-Chief and the Editorial Board. This follows the receipt of a notification from a reader, alerting the Editorial Board to a duplication present in the article in Figure 4A. Upon investigation, the Editorial Office identified further instances of high similarity. The specific duplication concerns involving Figure 4A are as follows:

- The second control panel (day 2) appears highly similar to the second STZ + Rubox istaurin panel (day 2) after a 90° rotation.
- The second control panel (day 2) appears highly similar to the fourth control panel (day 6) after a -11° rotation and a reduction in size.
- The second and fourth control panels appear highly similar to the Figure 3 GF group 15 Days panel after a 90° rotation from Rawaf et al. 2019 [retracted] (DOI: 10.1155/2019/4352470)
- The second and fourth control panels appear highly similar to the Figure 2A Normal Group 9-day panel after a 90° rotation from Yuan et al. 2018 [retracted] (DOI: 10.1042/BSR20171294), which is an article from the same authorship group.

The authors have been contacted with regard to the retraction and have not responded to the Journal's queries or the concerns. Given the extent of the issues raised, the Editorial Board stands by the decision to retract the article.

